# MYC Negative Rectal B-Cell Lymphoma, Unclassifiable, with Features Intermediate between Diffuse Large B-Cell Lymphoma and Burkitt's Lymphoma in an Immunocompetent Patient

**DOI:** 10.1155/2013/302304

**Published:** 2013-01-14

**Authors:** Jignesh G. Parikh, Ted Strom, Ilya Stone

**Affiliations:** ^1^Department of Pathology, University of Tennessee Health Science Center, Memphis, TN 38163, USA; ^2^Department of Pathology, Department of Veterans Affairs Medical Center, Memphis, TN 38104, USA

## Abstract

B-cell lymphoma, unclassifiable, with features intermediate between diffuse large B-cell lymphoma and Burkitt's lymphoma (BLUI) is a recently added entity to the World Health Organization (WHO) classification to address a grey zone between large B-cell lymphoma (DLBCL) and Burkitt's lymphoma (BL). These are rare aggressive lymphomas, which were previously also known as Burkitt's-like lymphoma (BLL). BL and BLUI/BLL of the colon mostly involve the ileocecal region. In the rectum, BL and BLUI/BLL almost always affect patients with the acquired immune deficiency syndrome (AIDS). We report the first case of rectal BLUI/BLL in an immunocompetent patient who is also negative for MYC abnormalities and the EBV early RNA (EBER) in situ hybridization.

## 1. Introduction

The border between classical Burkitt's lymphoma (BL) and classical diffuse large B-cell lymphoma (DLBCL) has been a field of diagnostic uncertainty [1]. The updated WHO classification of tumors of the hematopoietic and lymphoid tissues described these borderline lymphomas as “lymphoma, unclassifiable, with features intermediate between DLBCL and BL” (BLUI) [1]. Rectal BL and BLUI almost always affect patients with the human immunodeficiency virus (HIV) infection [2–4]. To the best of our knowledge, this is the first case of rectal BLUI in an immunocompetent patient who does not reveal MYC abnormalities and is also negative for EBV early RNA (EBER) in situ hybridization.

## 2. Case Report

**Figure 1:**
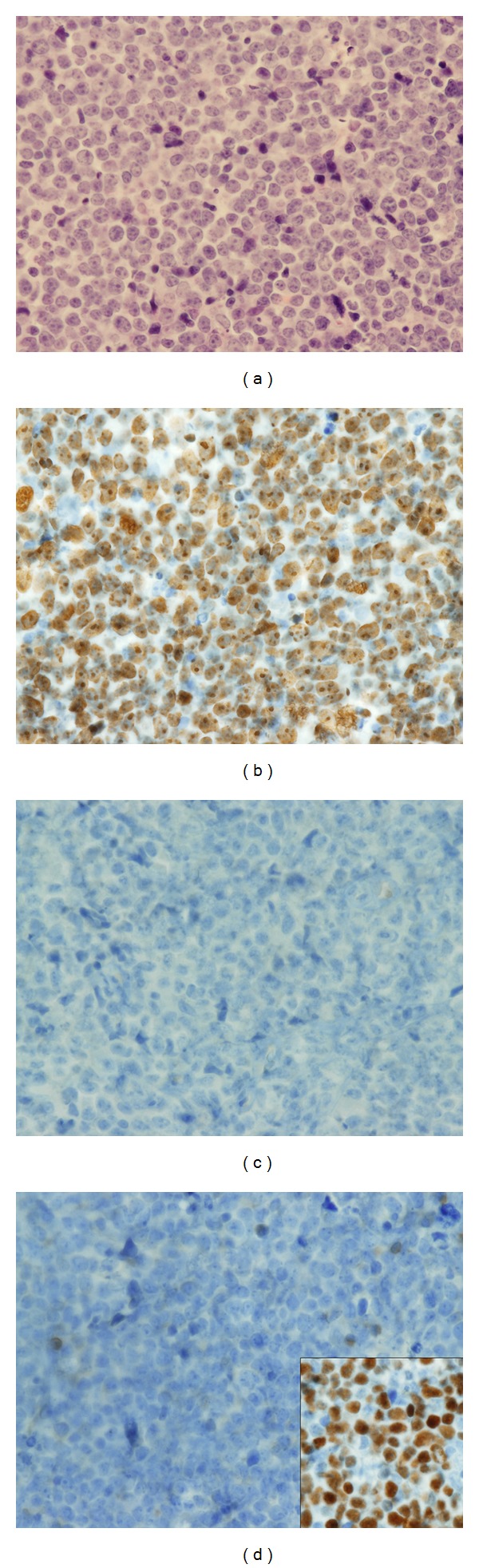
Histopathological evaluation. H&E stained sections show a monomorphic mucosal lymphoid proliferation with numerous mitotic figures (a). The lymphoid cells reveal positive immunoreactivity for Ki-67 (~100% positivity, (b)) and Bcl6 (inset, (d)); however, Bcl-2 immunostain was negative. EBER by in situ hybridization was also negative (c).

A sixty-three-year-old heterosexual male with a past history of aortic aneurysm and prostate cancer presented with hematochezia. On examination, a submucosal nodule measuring 6** **mm in greatest dimension was noted in the distal rectum about 2** **cm from the anal verge. Histopathological examination revealed a monomorphic mucosal lymphoid proliferation with numerous mitotic figures (Figure 1(a)). Nuclear polymorphism was slightly augmented compared to classic BL. The lymphoid cells expressed CD20, CD10, Bcl6 (Figure 1(d), inset), and Ki-67 (~100%, Figure 1(b)) and were negative for CD3, CD5, CD23, and Bcl2 (Figure 1(d)). EBER by in situ hybridization was also negative (Figure 1(c)). There was no morphologic, flow cytometric, or cytogenetic evidence of bone marrow involvement. Serologic tests for human immunodeficiency and hepatitis viruses were also negative. MYC translocations and rearrangement could not be detected by FISH. Based on nuclear pleomorphism and lack of MYC abnormalities, a diagnosis of rectal BLUI was made. Hematopathologists from a referral institute also concurred with this diagnosis.

## 3. Discussion

Among the gastrointestinal tract non-Hodgkins lymphomas (NHLs), primary gastric lymphomas are most common, whereas large intestinal lymphomas are rare [5]. With respect to the frequency, 62–80% involve the stomach, 13–31% involve the small intestine, and only 4–15% involve the large intestine [5]. The majority of large intestinal lymphomas occur in cecum, where a greater amount of lymphatic tissues are found [3]. Anorectal lymphomas represent only 3% of all gastrointestinal tract lymphomas. In contrast to their rare occurrence in the general population, the incidences of anorectal NHLs in patients with AIDS, particularly in homosexual patients, are very high [6].

BL is a rare aggressive form of NHL composed of monomorphic medium-sized B cells with basophilic cytoplasm and numerous mitotic figures. It is also characterized by c-MYC translocation and CD10+/bcl-2–/bcl-6+ with a very high Ki-67 proliferation index [7]. BL has been linked to AIDS, homosexuality, and EBV infection in the anorectum and in other organs [6, 7]. EBV in a latent form can be demonstrated by EBER in situ hybridization in the majority of BL patients [6, 7]. 

Morphology, immunophenotypic features, and gene expression studies suggest that BL and DLBCL are extreme ends of a spectrum of diseases [1, 8]. A group of lymphomas was characterized predominately by the fact that they are hard to assign to the one or the other group. These lesions in between both classical extremes have been described in the past as Burkitt's-like lymphoma (BLL), atypical BL, gray-zone lymphomas, B-cell lymphoma, unclassifiable, or borderline lymphoma [1]. These gray zone lymphomas have been recently termed “lymphoma, unclassifiable, with features intermediate between DLBCL and BL” (BLUI) by the updated WHO classification of tumors of the hematopoietic and lymphoid tissues [8].

We present a case of MYC negative rectal BLUI/BLL in an immunocompetent patient. A literature search through PubMed did not reveal any case of rectal BLUI/BLL in an immunocompetent patient. However, it revealed one case of rectal BL in an immunocompetent patient that was positive for EBER in situ hybridization (unlike the present case) [9]. Adult MYC negative BLUI are infrequent, and their clinical course is currently unknown [1]. At present, there is no established standard therapy for this entity [10]. A recent study suggests that MYC positive BLUI patients require more aggressive therapy than R-CHOP (rituximab plus cyclophosphamide, doxorubicin, vincristine, and prednisolone-related therapy) such as R-hyper-CVAD (rituximab plus hyperfractionated cyclophosphamide, vincristine, doxorubicin, and dexamethasone alternating with high-dose methotrexate and cytarabine) [10]. In contrast, MYC negative BLUI patients can be subdivided into two groups. One patient group is refractory to therapy, including intensive chemotherapy, and has a very poor survival. The second group responds to treatment (either R-CHOP or R-hyper-CVAD) with better survival [10]. Our patient was started on R-hyper-CVAD chemotherapy and is currently without any evidence of recurrence. 

Clinicians and pathologists must be aware of this rare aggressive entity while dealing with B-cell lymphomas of the rectum in immunocompetent patients.
